# 2-[(1*E*)-({[(Benzyl­sulfan­yl)methane­thio­yl]amino}­imino)­meth­yl]-6-meth­oxy­phenol: crystal structure and Hirshfeld surface analysis

**DOI:** 10.1107/S2056989016004291

**Published:** 2016-03-18

**Authors:** Enis Nadia Md Yusof, Mukesh M. Jotani, Edward R. T. Tiekink, Thahira B. S. A. Ravoof

**Affiliations:** a Department of Chemistry, Faculty of Science, Universiti Putra Malaysia, 43400, UPM Serdang, Selangor Darul Ehsan, Malaysia; b Department of Physics, Bhavan’s Sheth R. A. College of Science, Ahmedabad, Gujarat 380 001, India; c Research Centre for Crystalline Materials, Faculty of Science and Technology, Sunway University, 47500 Bandar Sunway, Selangor Darul Ehsan, Malaysia

**Keywords:** crystal structure, hydrogen bonding, di­thio­carbazate ester, Hirshfeld surface analysis

## Abstract

The title di­thio­carbazate ester, C_16_H_16_N_2_O_2_S_2_, comprises two almost planar residues, *i.e*. the phenyl ring and the remaining 14 non-H atoms (r.m.s. deviation = 0.0410 Å). These are orientated perpendicularly, forming a dihedral angle of 82.72 (5)°. An intra­molecular hy­droxy-O—H⋯N(imine) hydrogen bond, leading to an *S*(6) loop, is noted. An analysis of the geometric parameters is consistent with the mol­ecule existing as the thione tautomer, and the conformation about the C=N bond is *E*. The thione S and imine H atoms lie to the same side of the mol­ecule, facilitating the formation of inter­molecular N—H⋯S hydrogen bonds leading to eight-membered {⋯HNCS}_2_ synthons in the crystal. These aggregates are connected by phenyl-C—H⋯O(hy­droxy) inter­actions into a supra­molecular layer in the *bc* plane; these stack with no directional inter­actions between them. An analysis of the Hirshfeld surface confirms the nature of the inter­molecular inter­actions.

## Abstract 2

Two almost planar residues in the title thione are orientated perpendicularly [dihedral angle = 82.72 (5)°]; the conformation about the imine bond is *E*. In the crystal, centrosymmetric aggregates are formed *via* {⋯HNCS}_2_ synthons which are linked into supra­molecular layers by C—H⋯O inter­actions.

## Chemical context   

Di­thio­carbazate, NH_2_NHC(=S)S^−^, and more specifically substituted derivatives, have attracted the attention of researchers for decades (Ali & Livingstone, 1974 ▸). While a common motivation for investigating transition metal complexes of these anions relates to potential biological activity (Basha *et al.*, 2012 ▸; Vijayan *et al.*, 2015 ▸), including our own recent work (Yusof, Ravoof, Jamsari *et al.*, 2015 ▸; Yusof, Ravoof, Tiekink *et al.*, 2015 ▸), other motivations exist. Thus, recent studies have described the photo-catalytic production of hydrogen mediated by a conjugated nickel(II) bis-di­thio­carbazate complex (Wise *et al.*, 2015 ▸). The use of a coumarin-based di­thio­carbazate as a ratiometric and colormetric chemosensor for cobalt(II) is another recent development (Liu *et al.*, 2015 ▸). In rationalizing the electronic structures of metal di­thio­carbaza­tes, a knowledge of the uncomplexed or ‘free ligand’ structure is most useful. In keeping with this notion and as a part of an on-going study of the structural chemistry of metal di­thio­carbaza­tes and their ligands, the title compound was prepared and characterized both crystallographically and by a Hirshfeld surface analysis.

## Structural commentary   

**Figure 1:**
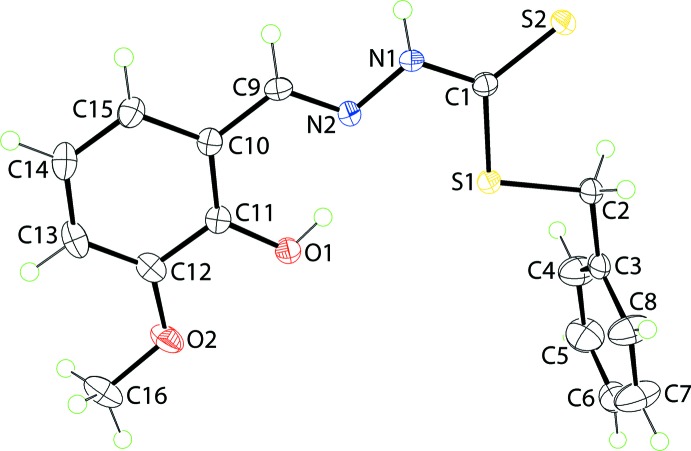
The mol­ecular structure of the title compound, showing the atom-labelling scheme and displacement ellipsoids at the 50% probability level.

The title compound, Fig. 1 ▸, comprises two almost planar regions, one being the phenyl ring, the other being the remaining 14 non-hydrogen atoms. The maximum deviations from the least-squares plane through the latter plane, with a r.m.s. deviation = 0.0410 Å, are 0.0715 (15) for the O1 atom and −0.0796 (18) for atom C16. To a first approximation, the mol­ecule can be described as having mirror symmetry with the 1,4-atoms of the terminal ring being bis­ected by the plane. Substanti­ating this description is the dihedral angle between the planes of 82.72 (5)°, indicating a very close to perpendicular relationship. The observed planarity in the larger fragment may be ascribed, in part, to the presence of an intra­molecular hy­droxy-O—H⋯N(imine) hydrogen bond (Table 1 ▸), which leads to the formation of an *S*(6) loop. The mol­ecule exists in the thione tautomeric form. Consistent with this assignment, the thione C1=S2 bond length, *i.e*. 1.670 (2) Å, is considerably shorter than the thiol C1—S1 and, especially, C2—S1 bonds of 1.749 (2) and 1.817 (2) Å, respectively. The conformation about the C=N bond is *E*, and the amine-N—H atom is flanked on either side by the thione-S and imine-H atoms.
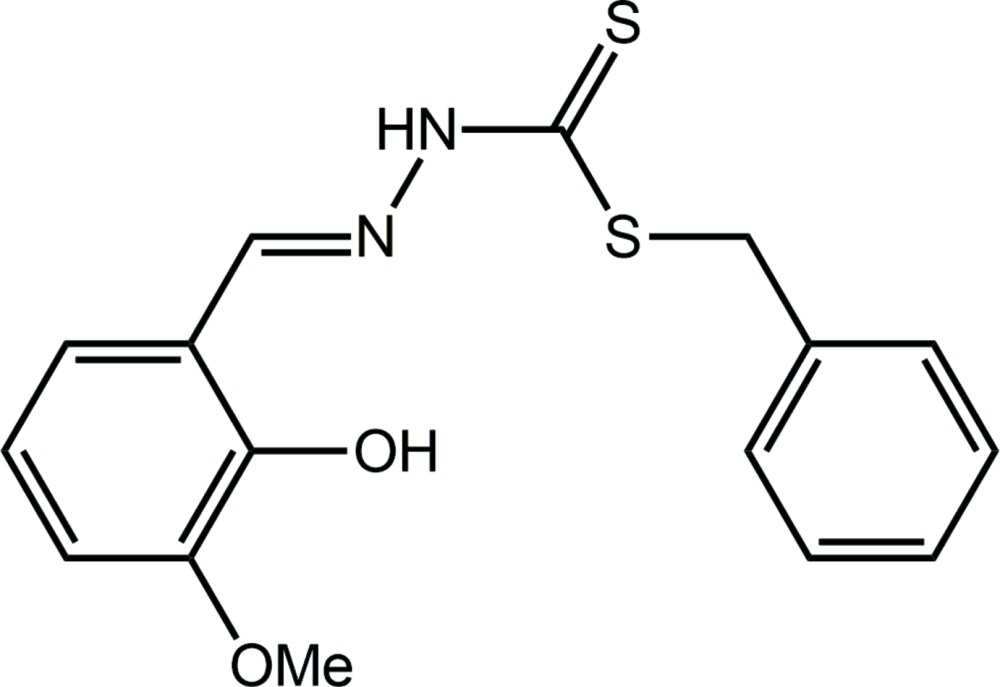


## Supra­molecular features   

**Figure 2:**
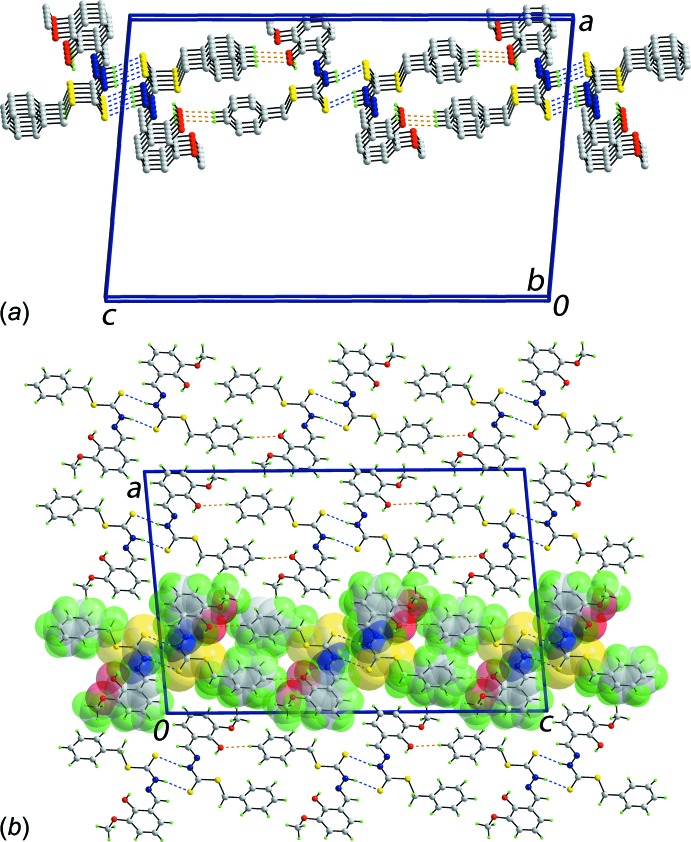
Mol­ecular packing in the title compound: (*a*) a perspective view of the supra­molecular layer sustained by thio­amide-N—H⋯S(thione) and phenyl-C—H⋯O(hy­droxy) inter­actions and, (*b*) a view of the unit-cell contents shown in projection down the *b* axis, highlighting one layer in space-filling mode. The N—H⋯S and C—H⋯O inter­actions are shown as blue and orange dashed lines, respectively. For (*a*), non-inter­acting H atoms have been omitted.

The most prominent feature of the packing of the title compound is the formation of centrosymmetric, eight-membered {⋯HNCS}_2_ synthons through the agency of thio­amide-N—H⋯S(thione) hydrogen bonds, Table 1 ▸. The dimeric aggregates are connected by phenyl-C—H⋯O(hy­droxy) inter­actions to form a supra­molecular layer in the *bc*-plane, Fig. 2 ▸ *a*. The layers stack along the *a* axis with no directional inter­actions between them, Fig. 2 ▸ *b.*

## Analysis of the Hirshfeld surfaces   

*Crystal Explorer 3.1* (Wolff *et al.*, 2012 ▸) was used to generate Hirshfeld surfaces mapped over *d*_norm_, *d*_e_, curvedness and electrostatic potential. The latter was calculated using *TONTO* (Spackman *et al.*, 2008 ▸; Jayatilaka *et al.*, 2005 ▸) which was integrated into *Crystal Explorer*; the experimental geometry was used as the input. Further, the electrostatic potentials were mapped on the Hirshfeld surface using the STO-3G basis set at the Hartree–Fock level of theory over the range ±0.1 au. The contact distances *d*_i_ and *d*_e_ from the Hirshfeld surface to the nearest atom inside and outside, respectively, enable the analysis of the inter­molecular inter­actions through the mapping of *d*_norm_. The combination of *d*_e_ and *d*_i_ in the form of a two-dimensional fingerprint plot (Rohl *et al.*, 2008 ▸) provides a summary of the inter­molecular contacts in the crystal.

**Figure 3:**
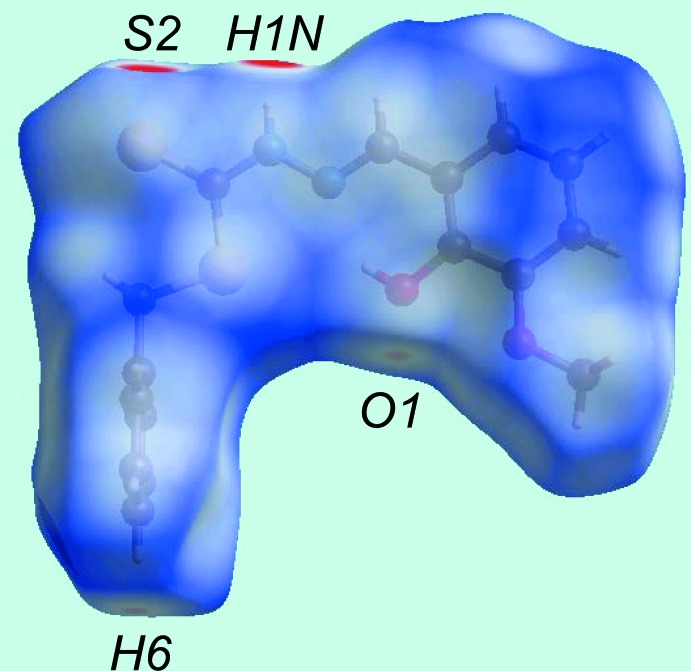
View of the Hirshfeld surface mapped over *d*_norm_.

**Figure 4:**
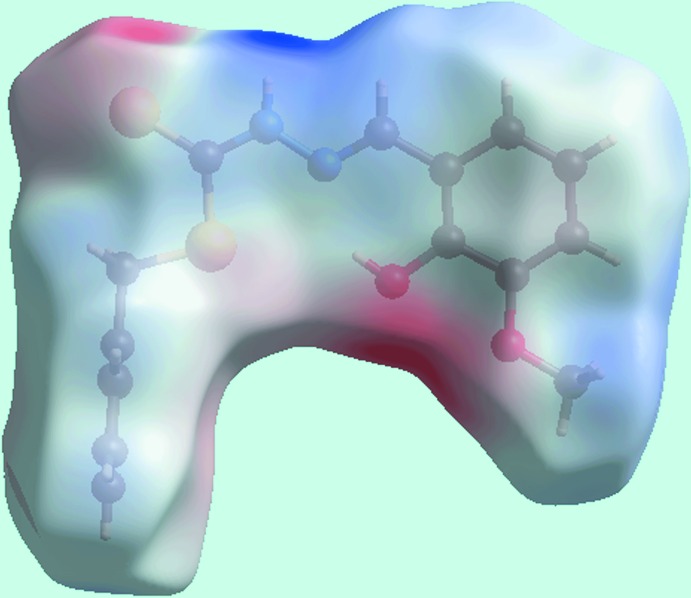
View of the Hirshfeld surface mapped over the electrostatic potential.

**Figure 5:**
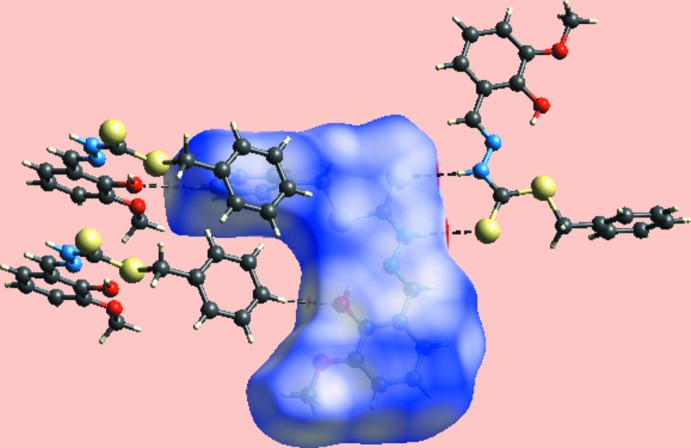
Hirshfeld surface mapped over *d*_norm_ showing hydrogen-bonding inter­actions with neighbouring mol­ecules.

From the view of the Hirshfeld surface mapped over *d*_norm_, Fig. 3 ▸, the deep-red depressions at atoms H1*N* and S2 confirm their role as the N—H⋯S hydrogen-bond donor and acceptor, respectively. On the surface mapped over the electrostatic potential, Fig. 4 ▸, these atoms appear as the respective blue and red regions. The light-red spots near the phenyl-hydrogen atom, H6, and hydroxyl oxygen, O1, on the *d*_norm_-mapped surface indicate the inter­molecular C—H⋯O inter­action between them. The immediate environment about the mol­ecule within *d*_norm_-mapped Hirshfeld surface mediated by the above inter­actions is illustrated in Fig. 5 ▸.

**Figure 6:**
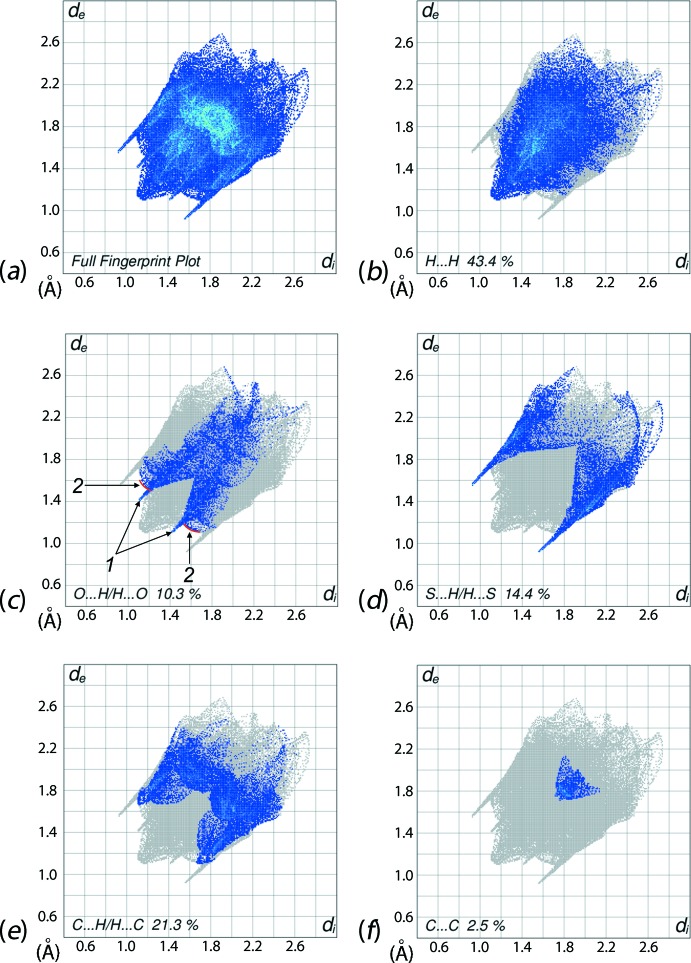
The two-dimensional fingerprint plots for (*a*) all inter­actions, and delineated into (*b*) H⋯H, (*c*) O⋯H/H⋯O, (*d*) S⋯H/H⋯S, (*e*) C⋯H/H⋯C and (*f*) C⋯C inter­actions.

**Figure 7:**
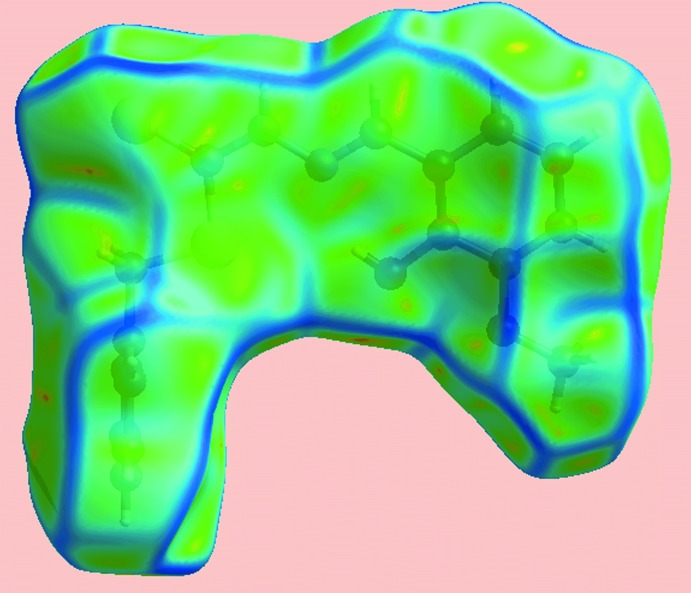
View of the Hirshfeld surface mapped over curvedness.

The overall two-dimensional fingerprint (FP) plot, Fig. 6 ▸ *a*, and those delineated into H⋯H, O⋯H/H⋯O, S⋯H/H⋯S, C⋯H/H⋯C and C⋯C inter­actions are illustrated in Fig. 6 ▸ *b*–*f*; the relative contributions are summarized in Table 2 ▸. The H⋯H contacts appear as the scattered points in nearly the entire plot, Fig. 6 ▸ *b*, and make a significant contribution, *i.e*. 43.4%, to the Hirshfeld surface. The round single peak at *d*_e_ + *d*_i_ ∼ 2.3 Å results from a short inter­atomic H⋯H contact, Table 3 ▸. The FP delineated into O⋯H/H⋯O contacts show a pair of short spikes at *d*_e_ + *d*_i_ ∼ 2.5 Å and the small arcs linked to them are identified with labels 1 and 2 in Fig. 6 ▸ *c*. These features correspond to a 10.3% contribution to the Hirshfeld surfaces and reflect the presence of inter­molecular C—H⋯O inter­actions as well as inter­atomic O⋯H contacts only slightly shorter than their van der Waals separation, *i.e*. around *d*_e_ + *d*_i_ ∼ 2.7 Å, Table 3 ▸. The presence of inter­molecular N—H⋯S hydrogen bonds in the crystal is evident from a prominent pair of sharp spikes in the outer region of the FP plot shown in Fig. 6 ▸ *d*, *i.e*. at *d*_e_ + *d*_i_ ∼ 2.45 Å, with a 14.4% contribution to the Hirshfeld surface. The distinct pair of wings corresponding to C⋯H/H⋯C contacts, Fig. 6 ▸ *e*, have ‘forceps-like’ tips at *d*_e_ + *d*_i_ ∼ 2.8 Å due to short inter­atomic C⋯H/H⋯C contacts, Table 3 ▸, although C—H⋯π inter­actions are not evident in the structure within the sum of their van der Waals radii. The 2.5% contribution from C⋯C contacts to the Hirshfeld surface features two overlapping triangles, Fig. 6 ▸ *f*, but the minimum (*d*_e_ + *d*_i_) distance is greater than van der Waals separation, confirming the absence of π–π stacking inter­actions. This is also evident from the small segments delineated by blue outlines in the Hirshfeld surface mapped over curvedness, Fig. 7 ▸.

The enrichment ratio (ER), based on Hirshfeld surface analysis, gives further description of inter­molecular inter­actions operating in a crystal (Jelsch *et al.*, 2014 ▸). The ER values are summarized in Table 4 ▸. The ER value close to but slightly less than unity, *i.e*. 0.97, for H⋯H contacts is in accord with expectation (Jelsch *et al.*, 2014 ▸). The sulfur atoms comprise 9.5% of Hirshfeld surface and the overall 14.4% contribution by S⋯H/H⋯S contacts results in an ER value of 1.14, which is in the expected range for N—H⋯S inter­actions, *i.e*. 1.0–1.5 (Jelsch *et al.*, 2014 ▸). The ER value of 1.28 corres­ponding to O⋯H/H⋯O contacts show a high propensity to form even though the percentage relative contribution to the overall surface, *i.e*. 10.3%, is small as is the 6.0% exposure provided by hydroxyl- and meth­oxy-oxygen atoms. The low ER value of 1.09 corres­ponding to C⋯C contacts is consistent with a low propensity for π–π stacking inter­actions in the structure. The presence of short inter­atomic non-bonded C⋯H/H⋯C contacts result in an ER value close to unity, Table 4 ▸, as there is little influence of C—H⋯π inter­actions on the mol­ecular packing. The other contributions to the surface *i.e*. N⋯H/H⋯N, S⋯S, S⋯O/O⋯S, C⋯S/S⋯C, *etc*. are very small and therefore, the ER values are not particularly informative although being > 1 for some inter­actions.

## Database survey   

Di­thio­carbazate *S*-esters are well studied with many examples included in the Cambridge Structural Database (Groom & Allen, 2014 ▸).
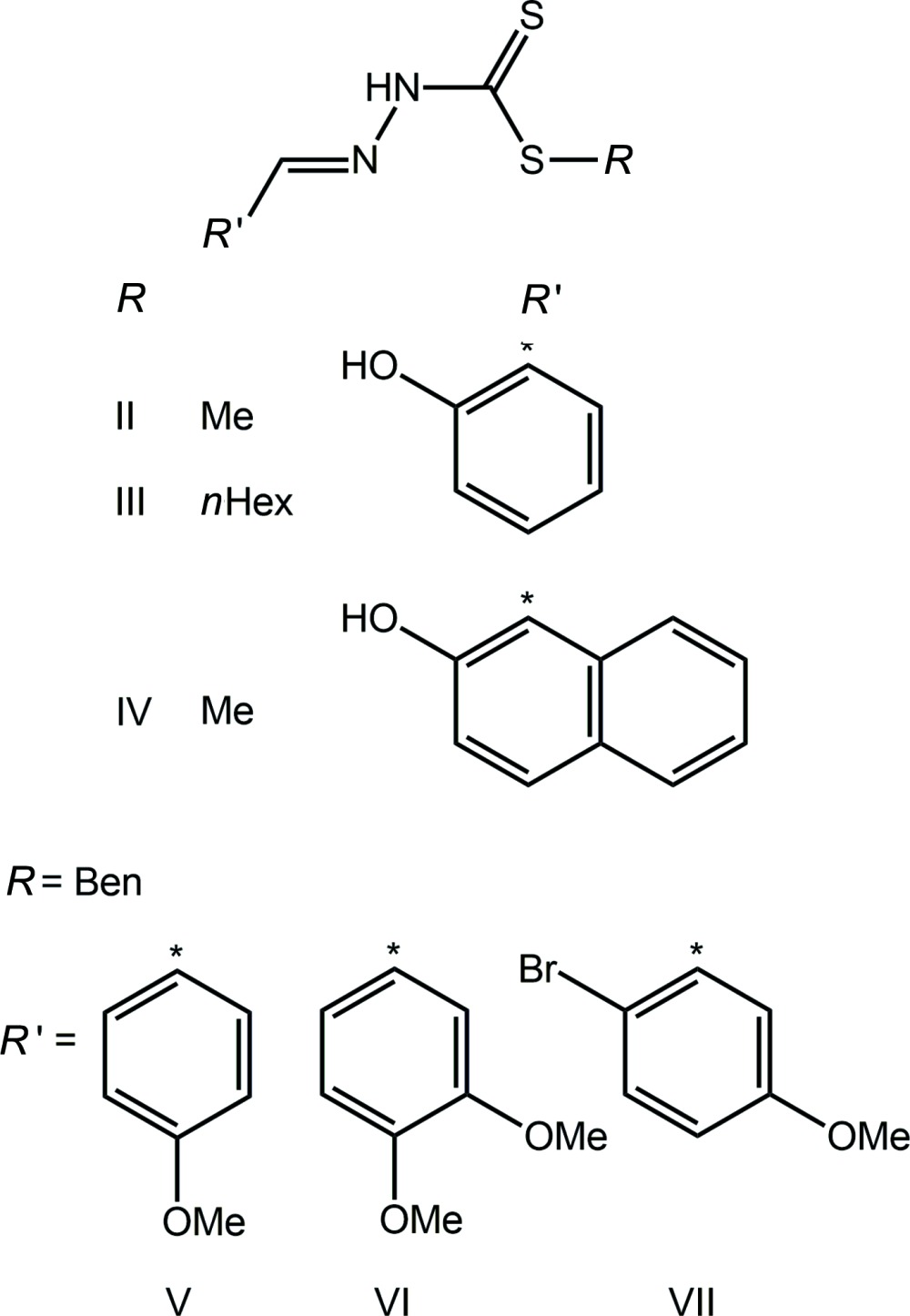


 There are three examples, II–IV, of derivatives with a hy­droxy group in the 2-position of the ring connected to the imine-C atom (Madanhire *et al.*, 2015 ▸; Sethuraman *et al.*, 2002 ▸; Begum *et al.*, 2016 ▸), with their chemical diagrams shown in Scheme 2. Inter­estingly, there are four virtually identical mol­ecules comprising the asymmetric unit in IV (Begum *et al.*, 2016 ▸). A common feature of each of I–IV, is the formation of an intra­molecular hy­droxy-O—H⋯N(imine) hydrogen bond. By contrast to I, each of the mol­ecules in II–IV is effectively planar. Reflecting the inter­est in the structural chemistry of this class of compound, there are several structures of *S*-benzyl esters with meth­oxy-substituted terminal rings (Fan *et al.*, 2011*a* ▸; Fan *et al.*, 2011*b* ▸; Tan *et al.*, 2015 ▸), see Scheme 2 for chemical structures. In each of V–VII, each meth­oxy group is co-planar with the benzene ring to which it is connected. To a first approximation, the mol­ecules in V and VII adopt approximately the same conformation as in I, *i.e*. with all but the benzyl rings lying in a plane. The dihedral angles between the two residues are 85.23 (12)° in V and 63.01 (8)° in VII. By contrast, a somewhat twisted conformation is observed for each of the two independent mol­ecules in VI.

## Synthesis and crystallization   

Following procedures adapted from the literature (Ali & Tarafder, 1977 ▸), *S*-benzyl­dithio­carbazate (SBDTC) (1.98 g, 0.01 mol) was dissolved in hot absolute ethanol (100 cm^3^) and added to an equimolar amount of 2-hy­droxy-3-meth­oxy­benzaldehyde (Merck, 1.52 g) in absolute ethanol (20 cm^3^). The mixture was heated with continuous stirring for about 30 min and was then allowed to stand overnight. The light-yellow crystals that formed were filtered and washed with absolute ethanol at room temperature. Yield: 70%. M.p.: 446–447 K. Analysis calculated for C_16_H_16_N_2_O_2_S_2_: C, 57.81; H, 4.85; N, 8.43. Found: C, 58.21; H, 4.87; N, 8.28%. FT–IR (ATR, cm^−1^): 3089, ν(N—H); 1598, ν(C=N); 1030, ν(N—N); 720, ν(C=S). ^13^C NMR (DMSO-*d*_6_) δ (p.p.m.): 196.09 (C=S), 148.59 (C=N); 147.45–114.43 (aromatic-C), 56.43 (CH_3_), 38.10 (CH_2_). *m*/*z* calculated for C_16_H_16_N_2_O_2_S_2_ 332.44, found 332.

## Refinement   

Crystal data, data collection and structure refinement details are summarized in Table 5 ▸. The carbon-bound H-atoms were placed in calculated positions (C—H = 0.95–0.99 Å) and were included in the refinement in the riding-model approximation, with *U*_iso_(H) set to 1.2*U*_eq_(C). The oxygen- and nitro­gen-bound H-atoms were located in a difference Fourier map but were refined with distance restraints of O—H = 0.84±0.01 Å and N—H = 0.88±0.01 Å, and with *U*_iso_(H) set to 1.5*U*_eq_(O) and 1.2*U*_eq_(N).

## Supplementary Material

Crystal structure: contains datablock(s) I, global. DOI: 10.1107/S2056989016004291/hb7572sup1.cif

Structure factors: contains datablock(s) I. DOI: 10.1107/S2056989016004291/hb7572Isup2.hkl

Supporting information file. DOI: 10.1107/S2056989016004291/hb7572Isup3.cml

CCDC reference: 1465209

Additional supporting information:  crystallographic information; 3D view; checkCIF report

## Figures and Tables

**Table 1:** Hydrogen-bond geometry (Å, °)

*D*—H⋯*A*	*D*—H	H⋯*A*	*D*⋯*A*	*D*—H⋯*A*
O1—H1*O*⋯N2	0.84 (2)	1.90 (2)	2.639 (2)	146 (2)
N1—H1*N*⋯S2^i^	0.88 (2)	2.47 (2)	3.3351 (18)	168 (2)
C6—H6⋯O1^ii^	0.95	2.51	3.453 (3)	170

Symmetry codes: (i) 
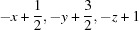
; (ii) 
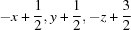
.

**Table 2:** Major percentage contribution of the different inter­molecular inter­actions to the Hirshfeld surface of the title compound

Contact	%
H⋯H	43.4
O⋯H/H⋯O	10.3
S⋯H/H⋯S	14.4
C⋯H/H⋯C	21.3
N⋯H/H⋯N	0.8
C⋯C	2.5
S⋯S	0.4
C⋯N/N⋯C	2.9
S⋯O/O⋯S	1.4
S⋯N/N⋯S	1.4
C⋯S/S⋯C	0.9

**Table 3:** Additional short inter­atomic contacts (Å) for the title compound

Inter­action	distance	symmetry operation
H8⋯H16*C*	2.25	− 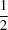 + *x*, 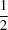 + *y*, *z*
C6⋯H16*B*	2.86	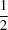 − *x*, 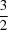 + *y*, 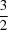 − *z*
O1⋯H16*A*	2.70	*x*, 1 + *y*, *z*
C11⋯H16*A*	2.78	*x*, 1 + *y*, *z*
C12⋯H16*A*	2.89	*x*, 1 + *y*, *z*

**Table 4:** Enrichment ratios (ER) for the title compound.

Inter­action	ER
H⋯H	0.97
O⋯H/H⋯O	1.28
S⋯H/H⋯S	1.14
C⋯C	1.09
C⋯H/H⋯C	1.05
S⋯O/O⋯S	1.24
S⋯S	0.45
C⋯S/S⋯C	0.31

**Table 5:** Experimental details

Crystal data	
Chemical formula	C_16_H_16_N_2_O_2_S_2_
*M*_r_	332.43
Crystal system, space group	Monoclinic, *C*2/*c*
Temperature (K)	100
*a*, *b*, *c* (Å)	20.7896 (10), 4.6965 (2), 32.5217 (13)
β (°)	95.004 (4)
*V* (Å^3^)	3163.3 (2)
*Z*	8
Radiation type	Mo *K*α
μ (mm^−1^)	0.35
Crystal size (mm)	0.25 × 0.15 × 0.07

Data collection	
Diffractometer	Agilent Xcalibur Eos Gemini
Absorption correction	Multi-scan (*CrysAlis PRO*; Agilent, 2011 ▸)
*T*_min_, *T*_max_	0.87, 0.98
No. of measured, independent and observed [*I* > 2σ(*I*)] reflections	6979, 3270, 2595
*R*_int_	0.033
(sin θ/λ)_max_ (Å^−1^)	0.628

Refinement	
*R*[*F* ^2^ > 2σ(*F* ^2^)], *wR*(*F* ^2^), *S*	0.041, 0.097, 1.06
No. of reflections	3270
No. of parameters	206
No. of restraints	2
H-atom treatment	H atoms treated by a mixture of independent and constrained refinement
Δρ_max_, Δρ_min_ (e Å^−3^)	0.33, −0.24

Computer programs: *CrysAlis PRO* (Agilent, 2011 ▸), *SHELXS97* (Sheldrick, 2008 ▸), *SHELXL2014* (Sheldrick, 2015 ▸), *ORTEP-3 for Windows* (Farrugia, 2012 ▸) and *DIAMOND* (Brandenburg, 2006 ▸), *publCIF* (Westrip, 2010 ▸).

## supplementary crystallographic information

### Crystal data

**Table:**

C_16_H_16_N_2_O_2_S_2_	*F*(000) = 1392
*M**_r_* = 332.43	*D*_x_ = 1.396 Mg m^−^^3^
Monoclinic, *C*2/*c*	Mo *K*α radiation, λ = 0.71073 Å
*a* = 20.7896 (10) Å	Cell parameters from 2185 reflections
*b* = 4.6965 (2) Å	θ = 2–29°
*c* = 32.5217 (13) Å	µ = 0.35 mm^−^^1^
β = 95.004 (4)°	*T* = 100 K
*V* = 3163.3 (2) Å^3^	Plate, light-yellow
*Z* = 8	0.25 × 0.15 × 0.07 mm

### Data collection

**Table:**

Agilent Xcalibur Eos Gemini diffractometer	3270 independent reflections
Radiation source: Enhance (Mo) X-ray Source	2595 reflections with *I* > 2σ(*I*)
Graphite monochromator	*R*_int_ = 0.033
Detector resolution: 16.1952 pixels mm^-1^	θ_max_ = 26.5°, θ_min_ = 2.4°
ω scans	*h* = −25→26
Absorption correction: multi-scan (*CrysAlis PRO*; Agilent, 2011)	*k* = −5→5
*T*_min_ = 0.87, *T*_max_ = 0.98	*l* = −27→40
6979 measured reflections	

### Refinement

**Table:**

Refinement on *F*^2^	2 restraints
Least-squares matrix: full	H atoms treated by a mixture of independent and constrained refinement
*R*[*F*^2^ > 2σ(*F*^2^)] = 0.041	*w* = 1/[σ^2^(*F*_o_^2^) + (0.0382*P*)^2^ + 1.7624*P*] where *P* = (*F*_o_^2^ + 2*F*_c_^2^)/3
*wR*(*F*^2^) = 0.097	(Δ/σ)_max_ = 0.002
*S* = 1.06	Δρ_max_ = 0.33 e Å^−^^3^
3270 reflections	Δρ_min_ = −0.24 e Å^−^^3^
206 parameters	

### Special details

**Table:**

Geometry. All esds (except the esd in the dihedral angle between two l.s. planes) are estimated using the full covariance matrix. The cell esds are taken into account individually in the estimation of esds in distances, angles and torsion angles; correlations between esds in cell parameters are only used when they are defined by crystal symmetry. An approximate (isotropic) treatment of cell esds is used for estimating esds involving l.s. planes.

### Fractional atomic coordinates and isotropic or equivalent isotropic displacement parameters (Å^2^)

**Table:**

	*x*	*y*	*z*	*U*_iso_*/*U*_eq_	
S1	0.22247 (2)	0.46299 (12)	0.61684 (2)	0.02185 (15)	
S2	0.17478 (3)	0.80851 (12)	0.54164 (2)	0.02258 (15)	
O1	0.35672 (7)	−0.0373 (3)	0.63252 (4)	0.0239 (3)	
H1O	0.3342 (10)	0.086 (4)	0.6194 (7)	0.036*	
O2	0.43677 (8)	−0.4115 (3)	0.66547 (5)	0.0305 (4)	
N1	0.27458 (8)	0.4630 (4)	0.54712 (5)	0.0203 (4)	
H1N	0.2819 (10)	0.519 (5)	0.5223 (4)	0.024*	
N2	0.31479 (8)	0.2682 (4)	0.56753 (5)	0.0194 (4)	
C1	0.22604 (9)	0.5777 (4)	0.56594 (6)	0.0179 (4)	
C2	0.15287 (10)	0.6579 (5)	0.63209 (6)	0.0237 (5)	
H2A	0.1579	0.8639	0.6268	0.028*	
H2B	0.1130	0.5907	0.6161	0.028*	
C3	0.14873 (10)	0.6059 (5)	0.67741 (6)	0.0223 (5)	
C4	0.18779 (13)	0.7499 (6)	0.70649 (8)	0.0395 (6)	
H4	0.2181	0.8838	0.6979	0.047*	
C5	0.18365 (13)	0.7029 (6)	0.74845 (8)	0.0432 (7)	
H5	0.2108	0.8059	0.7682	0.052*	
C6	0.14056 (12)	0.5086 (5)	0.76139 (7)	0.0356 (6)	
H6	0.1381	0.4735	0.7900	0.043*	
C7	0.10141 (14)	0.3670 (7)	0.73262 (8)	0.0502 (8)	
H7	0.0708	0.2346	0.7413	0.060*	
C8	0.10538 (13)	0.4127 (6)	0.69076 (8)	0.0404 (7)	
H8	0.0779	0.3096	0.6712	0.048*	
C9	0.35930 (10)	0.1553 (4)	0.54759 (6)	0.0192 (4)	
H9	0.3635	0.2118	0.5199	0.023*	
C10	0.40308 (9)	−0.0564 (4)	0.56664 (6)	0.0188 (4)	
C11	0.40002 (9)	−0.1423 (4)	0.60766 (6)	0.0191 (4)	
C12	0.44376 (10)	−0.3469 (5)	0.62508 (7)	0.0236 (5)	
C13	0.48986 (10)	−0.4610 (5)	0.60167 (7)	0.0282 (5)	
H13	0.5196	−0.5984	0.6134	0.034*	
C14	0.49292 (11)	−0.3747 (5)	0.56070 (7)	0.0286 (5)	
H14	0.5246	−0.4541	0.5448	0.034*	
C15	0.45042 (10)	−0.1762 (5)	0.54345 (7)	0.0239 (5)	
H15	0.4529	−0.1189	0.5156	0.029*	
C16	0.47583 (12)	−0.6366 (5)	0.68359 (8)	0.0346 (6)	
H16A	0.4679	−0.8109	0.6674	0.052*	
H16B	0.4649	−0.6693	0.7119	0.052*	
H16C	0.5215	−0.5840	0.6839	0.052*	

### Atomic displacement parameters (Å^2^)

**Table:**

	*U*^11^	*U*^22^	*U*^33^	*U*^12^	*U*^13^	*U*^23^
S1	0.0221 (3)	0.0271 (3)	0.0169 (3)	0.0071 (2)	0.0051 (2)	0.0040 (2)
S2	0.0225 (3)	0.0273 (3)	0.0180 (3)	0.0075 (2)	0.0024 (2)	0.0047 (2)
O1	0.0258 (8)	0.0281 (9)	0.0183 (7)	0.0072 (7)	0.0044 (6)	0.0034 (7)
O2	0.0399 (9)	0.0269 (9)	0.0238 (8)	0.0075 (7)	−0.0035 (7)	0.0064 (7)
N1	0.0206 (9)	0.0253 (10)	0.0154 (8)	0.0044 (8)	0.0040 (7)	0.0058 (8)
N2	0.0180 (8)	0.0208 (9)	0.0194 (9)	0.0039 (7)	0.0024 (7)	0.0026 (8)
C1	0.0181 (10)	0.0179 (10)	0.0176 (10)	−0.0018 (8)	0.0016 (8)	0.0000 (9)
C2	0.0239 (11)	0.0257 (12)	0.0222 (11)	0.0080 (9)	0.0061 (9)	0.0038 (10)
C3	0.0234 (11)	0.0231 (12)	0.0212 (11)	0.0093 (9)	0.0059 (9)	0.0005 (9)
C4	0.0430 (15)	0.0457 (16)	0.0293 (13)	−0.0093 (13)	0.0010 (12)	0.0040 (12)
C5	0.0523 (17)	0.0504 (17)	0.0258 (13)	−0.0002 (14)	−0.0034 (12)	−0.0029 (13)
C6	0.0411 (14)	0.0470 (16)	0.0200 (11)	0.0152 (12)	0.0103 (11)	0.0032 (12)
C7	0.0549 (18)	0.066 (2)	0.0310 (14)	−0.0188 (16)	0.0104 (13)	0.0078 (14)
C8	0.0493 (16)	0.0480 (16)	0.0244 (12)	−0.0172 (13)	0.0063 (12)	−0.0001 (12)
C9	0.0225 (10)	0.0203 (11)	0.0150 (10)	−0.0017 (9)	0.0028 (9)	0.0010 (9)
C10	0.0184 (10)	0.0170 (10)	0.0210 (10)	−0.0013 (9)	0.0015 (8)	−0.0030 (9)
C11	0.0185 (10)	0.0189 (11)	0.0197 (10)	−0.0013 (8)	0.0004 (9)	−0.0039 (9)
C12	0.0264 (11)	0.0194 (11)	0.0239 (11)	−0.0004 (9)	−0.0046 (9)	−0.0011 (9)
C13	0.0239 (11)	0.0238 (12)	0.0355 (13)	0.0068 (10)	−0.0067 (10)	−0.0046 (11)
C14	0.0228 (11)	0.0301 (13)	0.0331 (13)	0.0063 (10)	0.0035 (10)	−0.0077 (11)
C15	0.0223 (11)	0.0269 (12)	0.0228 (11)	0.0012 (9)	0.0041 (9)	−0.0030 (10)
C16	0.0411 (14)	0.0237 (13)	0.0363 (14)	−0.0008 (11)	−0.0129 (12)	0.0070 (11)

### Geometric parameters (Å, º)

**Table:**

S1—C1	1.749 (2)	C6—C7	1.359 (4)
S1—C2	1.817 (2)	C6—H6	0.9500
S2—C1	1.670 (2)	C7—C8	1.388 (3)
O1—C11	1.354 (2)	C7—H7	0.9500
O1—H1O	0.836 (10)	C8—H8	0.9500
O2—C12	1.368 (3)	C9—C10	1.450 (3)
O2—C16	1.429 (3)	C9—H9	0.9500
N1—C1	1.338 (2)	C10—C11	1.401 (3)
N1—N2	1.371 (2)	C10—C15	1.408 (3)
N1—H1N	0.874 (9)	C11—C12	1.408 (3)
N2—C9	1.290 (2)	C12—C13	1.383 (3)
C2—C3	1.504 (3)	C13—C14	1.400 (3)
C2—H2A	0.9900	C13—H13	0.9500
C2—H2B	0.9900	C14—C15	1.370 (3)
C3—C4	1.370 (3)	C14—H14	0.9500
C3—C8	1.376 (3)	C15—H15	0.9500
C4—C5	1.392 (3)	C16—H16A	0.9800
C4—H4	0.9500	C16—H16B	0.9800
C5—C6	1.370 (4)	C16—H16C	0.9800
C5—H5	0.9500		
			
C1—S1—C2	101.75 (10)	C3—C8—C7	120.5 (2)
C11—O1—H1O	108.6 (16)	C3—C8—H8	119.7
C12—O2—C16	117.13 (17)	C7—C8—H8	119.7
C1—N1—N2	119.96 (16)	N2—C9—C10	121.21 (18)
C1—N1—H1N	120.0 (15)	N2—C9—H9	119.4
N2—N1—H1N	120.0 (15)	C10—C9—H9	119.4
C9—N2—N1	117.70 (17)	C11—C10—C15	119.1 (2)
N1—C1—S2	121.37 (15)	C11—C10—C9	121.72 (18)
N1—C1—S1	113.89 (15)	C15—C10—C9	119.15 (18)
S2—C1—S1	124.74 (11)	O1—C11—C10	123.47 (19)
C3—C2—S1	107.49 (14)	O1—C11—C12	116.60 (18)
C3—C2—H2A	110.2	C10—C11—C12	119.92 (18)
S1—C2—H2A	110.2	O2—C12—C13	125.4 (2)
C3—C2—H2B	110.2	O2—C12—C11	114.77 (18)
S1—C2—H2B	110.2	C13—C12—C11	119.8 (2)
H2A—C2—H2B	108.5	C12—C13—C14	120.2 (2)
C4—C3—C8	118.2 (2)	C12—C13—H13	119.9
C4—C3—C2	121.0 (2)	C14—C13—H13	119.9
C8—C3—C2	120.8 (2)	C15—C14—C13	120.4 (2)
C3—C4—C5	121.0 (2)	C15—C14—H14	119.8
C3—C4—H4	119.5	C13—C14—H14	119.8
C5—C4—H4	119.5	C14—C15—C10	120.6 (2)
C6—C5—C4	120.3 (3)	C14—C15—H15	119.7
C6—C5—H5	119.8	C10—C15—H15	119.7
C4—C5—H5	119.8	O2—C16—H16A	109.5
C7—C6—C5	118.8 (2)	O2—C16—H16B	109.5
C7—C6—H6	120.6	H16A—C16—H16B	109.5
C5—C6—H6	120.6	O2—C16—H16C	109.5
C6—C7—C8	121.1 (3)	H16A—C16—H16C	109.5
C6—C7—H7	119.4	H16B—C16—H16C	109.5
C8—C7—H7	119.4		

### Hydrogen-bond geometry (Å, º)

**Table:**

*D*—H···*A*	*D*—H	H···*A*	*D*···*A*	*D*—H···*A*
O1—H1*O*···N2	0.84 (2)	1.90 (2)	2.639 (2)	146 (2)
N1—H1*N*···S2^i^	0.88 (2)	2.47 (2)	3.3351 (18)	168 (2)
C6—H6···O1^ii^	0.95	2.51	3.453 (3)	170

Symmetry codes: (i) −*x*+1/2, −*y*+3/2, −*z*+1; (ii) −*x*+1/2, *y*+1/2, −*z*+3/2.
